# Mouse IgA modulates human gut microbiota with inflammatory bowel disease patients

**DOI:** 10.1007/s00535-024-02121-y

**Published:** 2024-06-14

**Authors:** Keishu Takahashi, Naoki Morita, Ryutaro Tamano, Peng Gao, Noriho Iida, Akira Andoh, Hirotsugu Imaeda, Ken Kurokawa, Mayo Tsuboi, Yoku Hayakawa, Mitsuhiro Fujishiro, Reiko Shinkura

**Affiliations:** 1 Institute for Quantitative Biosciences, The University of Tokyo, Bunkyo-ku, Tokyo Japan Laboratory of Immunology and Infection Control,; 2 Graduate School of Frontier Science, The University of Tokyo, Kashiwa, Chiba Japan; 3 Department of Gastroenterology, Graduate School of Medical Sciences, Kanazawa University, Kanazawa, Ishikawa Japan; 4 Department of Medicine, Shiga University of Medical Science, Otsu, Shiga Japan; 5 Department of Gastroenterology, Nagahama City Hospital, Nagahama, Shiga Japan; 6 Department of Gastroenterology, Graduate School of Medicine, The University of Tokyo, Bunkyo-ku, Tokyo Japan; 7 Collaborative Research Institute for Innovative Microbiology, The University of Tokyo, Bunkyo-ku, Tokyo Japan

**Keywords:** Inflammatory bowel disease, IgA, Gut microbiota, Dysbiosis

## Abstract

**Background:**

The imbalance of commensal bacteria is called dysbiosis in intestinal microflora. Secreted IgA in the intestinal lumen plays an important role in the regulation of microbiota. Although dysbiosis of gut bacteria is reported in IBD patients, it remains unclear what makes dysbiosis of their microflora. The intervention method for remedy of dysbiosis in IBD patients is not well established. In this study, we focused on the quality of human endogenous IgA and investigated whether mouse monoclonal IgA which binds to selectively colitogenic bacteria can modulate human gut microbiota with IBD patients.

**Methods:**

IgA-bound and -unbound bacteria were sorted by MACS and cell sorter. Sorted bacteria were analyzed by 16S rRNA sequencing to investigate what kinds of bacteria endogenous IgA or mouse IgA recognized in human gut microbiota. To evaluate the effect of mouse IgA, gnotobiotic mice with IBD patient microbiota were orally administrated with mouse IgA and analyzed gut microbiota.

**Results:**

We show that human endogenous IgA has abnormal binding activity to gut bacteria in IBD patients. Mouse IgA can bind to human microbiota and bind to selectively colitogenic bacteria. The rW27, especially, has a growth inhibitory activity to human colitogenic bacteria*.* Furthermore, oral administration of mouse IgA reduced an inflammation biomarker, fecal lipocalin 2, in mice colonized with IBD patient-derived microbiota, and improved dysbiosis of IBD patient sample.

**Conclusion:**

Oral treatment of mouse IgA can treat gut dysbiosis in IBD patients by modulating gut microbiota.

**Supplementary Information:**

The online version contains supplementary material available at 10.1007/s00535-024-02121-y.

## Introduction

Recent multitudes of studies uncovered that intestinal commensal bacteria play an important role in the maintenance of homeostasis in the host [1]. Disorder of healthy gut bacteria is associated with many diseases including inflammatory bowel disease (IBD) [2, 3]. These conditions of microbiota are called dysbiosis. It has been suggested that a change in microbiota affects the function of host cells through microbiota-derived metabolites [4, 5]. The intestinal microbiota is also influenced by lifestyle, antibiotics, and aging [6, 7]. Many researchers try to recover from dysbiosis to healthy microbiota to improve disease severity. However, succeeded trial was limited.

IBD is consisted by Crohn’s disease (CD) and ulcerative colitis (UC) which is associated with intensive and chronic inflammation in the intestinal tract. Accumulating evidence suggests that environmental factors including gut bacteria play a pivotal role in the pathogenesis of IBD [2, 3]. Many of the research illustrated that specifically noted gut bacteria act as drivers for the initiation of host inflammatory responses.

Intestinal cell-derived factors such as Immunoglobulin A (IgA) work on the microbiota to maintain healthy conditions of the intestine. It is well known that IgA is the most abundant antibody isotype in human and mainly secreted into the intestinal lumen from gut lamina propria [8]. Mucosal IgA is produced by IgA-producing plasma cells associated with class switching recombination and somatic hypermutation to introduce mutations in variable regions of antibodies through activation-induced cytidine deaminase (AID) [9, 10]. Intestinal IgA, which is mainly a homodimer, is secreted into lumen via poly-Ig receptor, which is expressed in intestinal epithelial cells [11, 12]. Secreted IgA recognizes intestinal commensal bacteria to maintain gut homeostasis. However, dysfunction of IgA is associated with dysbiosis [13, 14]. It is also reported that insufficient secreted IgA is one of the causes of IBD [15, 16].

In previous reports, we identified mouse-derived W27 monoclonal IgA antibody [17–19]. W27 binds to multiple pathogenic bacteria such as *Escherichia (E.) coli*, but not *Lacticaseibacillus (L.) casei*, which is one of the representative beneficial bacteria. W27 has a function to inhibit the cell growth of *E. coli* but not *L. casei*, indicating that W27 selectivity acts on pathogenic bacteria*.* This function raises a possibility that oral treatment of W27 modifies gut microbiota with dysbiosis toward healthy ones by selective elimination of pathogenic bacteria, as reported previously in mouse models [17, 18].

In this study, we demonstrated that the aberrant binding ability of intestinal IgA derived from IBD patients and that selected mouse-derived monoclonal IgA clones bind to candidates of colitogenic bacteria in IBD patients, including *E. coli, Enterococcus* (*E.*) *faecium, Gemella* (*G.*) *morbillorum* and *Veillonella* (*V*.) *dispar.* According to IgA binding to these bacteria, a recombinant (r) W27 IgA suppressed those bacterial growth in vitro. Moreover, oral treatment of rW27 increased the diversity of gut microbiota in IBD patient-derived fecal transplanted mice. Thus, mouse IgA can bind to human microbiota and ameliorate dysbiosis of microbiota from IBD patients.

## Methods

### Subjects

Fecal samples were provided by healthy Japanese volunteers (*n* = 12) and patients diagnosed as UC and CD (one sample per subject). Information on IBD patients (*n* = 20, UC: *n* = 13, CD: n = 7) and healthy controls (HC, *n* = 12) is indicated in Table 1 and Supplementary Table 1.

**Table 1:** Information of Healthy controls and IBD patients

	Healthy controls (*n* = 12)	IBD patients (*n* = 20)	*P* value
Gender (male/female)	3/9	16/5	0.009
Age (years), median (min–max)	49 (26–64)	39 (21–73)	0.170
BMI (kg/m^2^), median (min–max)	21 (19–24)	20 (16–27)	0.894

	Ulcerative Colitis (UC)/Crohn's Disease (CD)	13/7
UC	Pan Colitis	10
	Left-sided colitis	3
	Mayo score, median (min–man)	8 (0–11)
CD	Ileocolon	4
	Ileum	1
	Colon	2
	CDAI, median (min–max)	123 (40–303)
	C-reactive protein (mg/dl), median (min–max)	3.20 (0.02–14.90)
	Albumin (mg/dl)	3.10 (2.00–5.00)
	Hemoglobin (g/dl), median (min–max)	13.05 (8.00–15.20)
Treatment		Yes/No
5-Aminosalicylic acid		16/4
Prednisolone		8/12
AZA/6-MP		2/18
Tac/CyA		1/19
Infliximab		4/16
Adalimumab		2/18

Statistical analysis was performed by chi-squared test

*BMI* body mass index, *AZA/6-MP* azathioprine/6-mercaptopurine, *Tac/CyA* tacrolimus/ciclosporin

### Ethics Statement

The study protocol of all enrolled participants was approved by the ethics committee of Shiga University of Medical Science (25–182), Kanazawa University (2016–450), and The University of Tokyo (10,329-(11)). All participants were assigned informed consent. All subjects signed a consent form to allow us to publish our findings.

#### ELISA

The concentration of antibody was measured by sandwich ELISA. Capture antibody (2 μg/ml) was suspended in 0.05 M Na_2_CO_3_ and was coated into ELISA plates (Thermo Fisher). Capture antibodies were goat anti-mouse IgA, anti-human IgA or IgG (Southern Biotech). After blocking by 1% bovine serum albumin (Wako) in PBS, the concentration of antibody was detected with alkaline phosphatase (AP)-conjugated antibody and AP substrate (Sigma). Mouse IgA was detected with AP-conjugated goat anti-mouse IgA (Southern Biotech). Human antibody was detected with AP-conjugated goat anti-human IgA or IgG (Southern Biotech). After adding substrate solution, the optical density (O.D.) 405 nm values were measured by Tristar2 LB942 (BERTHOLD TECHNOLOGIES).

### Purification of recombinant mouse IgA

According to the protocol of the ExpiCHO Expression System (Thermo Scientific), W27 heavy, W27 light, and J chain gene-containing pcDNA3.1 ( +) vectors were introduced into Chinese hamster ovary cells. The culture supernatant was collected and passed through 0.22 μm filter (Thermo Scientific). Since the previously reported W27 IgA [17] produced by hybridoma does not bind to the protein L column (Cytiva), several amino acid mutations were introduced into the light chain to purify the recombinant (r) W27 by protein L column. In the same manner as the rW27, recombinant IgA antibodies (rPG151A, rPG160A) were purified by a protein L column. The concentration of purified IgA was measured by sandwich ELISA.

### Purification of mouse IgA derived from IgA-producing hybridoma

IgA-producing hybridoma was generated as described previously [17]. In brief, isolated intestinal lamina propria cells which derived from mice were fused with NS-1 cells with polyethylene glycol. The cell fusion and the subcloning method were followed by the manufacturer’s protocol (ClonaCell-HY Hybridoma Cloning Kit, STEMCELL Technologies). IgA-producing hybridomas were selected by detection of IgA in the culture supernatant by sandwich ELISA. In this study, W37, PGSI1A, and SPFSI12 were derived from IgA-producing hybridoma. Culture supernatants of IgA-producing hybridoma were also purified with the protein L column. The concentration of purified IgA was measured by sandwich ELISA.

### Biotinylation of purified mouse-derived monoclonal IgA

The purified mouse IgA was biotinylated according to the EZ-Link NHS-LC-Biotin (Thermo Scientific) protocol. Biotinylated antibodies were dialyzed with Slide A Lyzer Dialysis Cassette G2 (Thermo Scientific) and sterilized through a 0.22 μm filter (BMBio). The concentration of biotinylated-mouse IgA was measured by sandwich ELISA.

### Preparation of human fecal samples

Human stool samples were weighted, suspended in 9 volumes of PBS, and centrifuged (50 g, 15 min, 4 °C) to remove large particles under anaerobic conditions (80% N_2_, 10% H_2_, 10% CO_2_) in an anaerobic chamber (Coy Laboratory Products). Supernatant of stool sample was collected. Well-suspended samples were aliquoted 1 ml and stored at -80 °C until use. Dissolved samples were centrifuged (8,000 g, 5 min, 4 °C). Supernatants were filtered and measured the concentration of fecal antibodies by sandwich ELISA.

### Analysis of human endogenous and mouse IgA-bound *bacteria* by flow cytometry

Bacterial numbers in human fecal samples or cultured bacteria were calculated by following the protocol of Cell Viability Kit (BD). After counting the bacteria number, bacteria were washed with PBS (8,000 g, 5 min, 4 °C). Bacteria were incubated with 20% normal rat serum (Wako) for blocking for 30 min on ice. For detection of human endogenous IgA, 6 × 10^6^ cells were reacted with goat anti-human IgA-PE (Southern Biotech) for 30 min on ice. For detection of mouse IgA, 6 × 10^6^ cells were reacted with 15 μg of biotinylated-mouse IgA for 30 min on ice and after washed, reacted with streptavidin-PE/Cy7 (Biolegend) for 30 min on ice. Thereafter, bacteria were washed with 10% fetal bovine serum and EDTA-containing PBS (FACS) buffer (8,000 g, 5 min, 4 °C). Bacteria were reacted with Thiazole Orange (TO, BD) and the percentage of human endogenous or mouse IgA-bound bacteria was analyzed by SONY Cell Sorter SH800 (SONY).

### Sorting for IgA-bound and -unbound *bacteria* by magnetic cell sorting (MACS) and cell sorter

Bacterial numbers were calculated as described above. After blocking, for human endogenous IgA, 6 × 10^6^ cells were reacted with 15 µl of anti-human IgA-PE antibody (Milteny) for 30 min on ice. Bacteria were washed with FACS buffer (8,000 g, 5 min, 4 °C) and were reacted with 15 µl of Anti-PE Microbeads (Milteny) for 30 min on ice. For mouse IgA, 6 × 10^6^ cells were reacted with 15 μg of biotinylated-mouse IgA for 30 min on ice. Bacteria were washed with FACS buffer (8,000 g, 5 min, 4 °C) and were reacted with 15 µl of Microbeads-Streptavidin (Milteny) for 30 min on ice. After reaction, IgA-bound and -unbound bacteria were sorted by MACS according to LS column protocol (Milteny). Human endogenous IgA-bound and -unbound bacteria were stained by TO (BD) for 20 min on ice. Mouse IgA-bound and -unbound bacteria were stained by TO (BD) and goat anti-mouse IgA-PE (Southern Biotech) for 20 min on ice. IgA-bound and -unbound bacteria fractions were separated by SONY Cell Sorter SH800 and confirmed that a recovery ratio was more than 80%.

### Bacterial DNA extraction

Bacterial genomic DNA extraction was performed according to a previous report [17] with minor modification. In brief, bacteria were lysed in DNA Lysis buffer (50 mM Tris–HCl, 300 mM NaCl, 1 mM EDTA and 0.5% SDS) on bead smash with glass beads (GB-01, TOMY) and incubated with proteinase K (0.2 mg/ml, nacalai) overnight at 55 °C. Bacterial DNA was purified by phenol/chloroform/isoamyl alcohol extraction and ethanol precipitation. Finally, DNA was dissolved in ultra-pure distilled water (Invitrogen) and stored at -20 °C.

### Sequencing and analysis of 16S rRNA

Bacterial DNA was amplified by polymerase chain reaction (PCR) targeting the V3-V4 variable region of 16S ribosomal RNA (rRNA) gene with the following program: preheating at 98 °C for 3 min; 30 cycles of denaturation at 98 °C for 10 s, annealing at 46 °C for 5 s and extension at 72 °C for 30 s; and a terminal extension at 72 °C for 5 min. The primers used for 16S rRNA sequence were as follows: 342Fw (5’-AATGATACGGCGACCACCGAGATCTACACTCTTTCCCTACACGACGCTCTTCCGATCTCTACGGGGGGCAGCAG-3’), 806Rv (5’- CAAGCAGAAGACGGCATACGAGATXXXXXXGTGACTGGAGTTCAGACGTGTGCTCTTCCGATCTGGACTACCGGGGTATCT-3’). XXXXXX represents the index nucleotide sequence. PCR product was purified with Fast Gene gel extraction kit (Nippon Genetics). Sequence analysis was performed by Myskin Corporation using Miseq Reagent Kit V3 (Illumina). The sequence data were analyzed by Quantitative Insights into Microbial Ecology (Qiime) 2 software (ver. 2020–2). Denoising and trimming of sequences were used by DADA2. The first 16 bp and 17 bp were trimmed from the 5’ end of both forward and reverse reads to remove primer sequences. Taxonomic assignment was determined with the Greengenes and SILVA [20, 21]. For the α-diversity, Shannon index was calculated by Qiime2. For the β-diversity, principal coordinate analysis based on unweighted unifrac distance (PERMANOVA/adonis) was analyzed by QIIME2 and the vegan package for R (version 2.6–4). The heatmap analysis was performed by R (version 4.2.3). The differences in each group were analyzed by linear discriminant analysis effect size (Lefse) analysis [22].

### Calculation of IgA index

The calculation of IgA index was performed following previous reports [23, 24] with some modifications. IgA binding ability is the ratio of IgA-bound bacteria to gut microbiota. Since IgA binding ability of the individual is different, IgA _taxon abundance_ was calculated based on the relative abundance of each fraction. This was used to calculate the ratio of IgA-bound or -unbound bacteria (IgA^+^_taxon abundance_, IgA^−^_taxon abundance_) for each individual, followed by the IgA index calculation. In this study, bacteria with median value of IgA index greater than 0 are considered to be strongly bound by IgA.

$${[\text{log}\left({\text{IgA}}_{\text{taxon abundance}}^{+}\right)-log\left({\text{IgA}}_{\text{relative abundance}}^{+}\times \text{IgA binding ability}/100\right)]}$$

$${[\text{log}\left({\text{IgA}}_{\text{taxon abundance}} ^{+}\right)-log\left({\text{IgA}}_{\text{relative abundance}}^{-}\times (1-\text{IgA binding ability}/100)\right)]}$$

$$\text{IgA index}=\frac{[\text{log}\left({\text{IgA}}_{{\text{taxon abundance}}}^{+}\right)-log\left({\text{IgA}}_{\text{taxon abundance } }^{-}\right)]}{[\text{log}\left({\text{IgA}}_{{\text{taxon abundance}}}^{+}\right)+log\left({\text{IgA}}_{\text{taxon abundance }}^{-}\right)]}$$

### Culture condition of *bacteria*

Bacteria were cultured at 37 °C under aerobic condition or anaerobic condition (80% N_2_, 10% H_2_, 10% CO_2_) in an anaerobic chamber. *E. coli* (BW38029) was cultured in Lysogeny Broth (LB) medium (nacalai) in aerobic conditions. *Clostridioides difficile* (*C. difficile*, ATCC10463) and *E. faecium* (ATCC19434) were cultured in Brain Heart Infusion (BHI) medium (BD) in anaerobic condition. *G. morbillorum* (ATCC27824) was cultured in Tryptic soy broth (BD) containing 5% sheep-defibrinated blood (Japan Bioceram) in anaerobic condition. *V. dispar* (ATCC 17748) was cultured in BHI medium containing hemin (Tokyo Chemical industry, 50 µg/ml) and vitamin K1 (nacalai, 0.15 µg/ml) in anaerobic condition. All bacteria were picked up from a glycerol stock and cultured in each medium overnight at 37 °C.

### ELISA for mouse IgA binding assay against *bacteria*

After the cultivation of all bacteria, bacteria were washed with PBS (8,000 g, 5 min, 4 °C). Bacteria were suspended in 0.05 M Na_2_CO_3_ and were coated on ELISA plates (Thermo Fisher) at a concentration of 1 × 10^8^ cells/well. After blocking, the relative binding ability of mouse IgA was detected with AP-conjugated goat anti-mouse IgA (Southern Biotech) as previously mentioned.

### Growth inhibition analysis for *bacteria*

All bacteria were cultured in appropriate conditions described above. For *E. coli*, after cultured, they diluted to 1/30,000 with LB medium. The 5 µl of bacterial solution (approximately 300 cells/tube) was added to 25 µl of PBS or rW27 (1 mg/ml), and the mixture was incubated at 37 °C for 2 h. Then, 30 µL of the culture medium was added and further incubated at 37 °C for 3 h. For other bacteria, after counting bacteria, they were diluted to 1 × 10^4^ cells/µl of *E. faecium*, 2 × 10^3^ cells/µl of *G. morbillorum* and *V. dispar* with appropriate culture medium. The 5 µl of bacterial solution was added to 25 µl of PBS or rW27 (1 mg/ml), and the mixture was cultured at 37 °C for 1 h. Then, 30 µL of the culture medium was added and cultured at 37 °C for 8 h. After incubation, bacteria were seeded on each agar plates, and cultured at 37 °C overnight.

### Animals

Mice were kept and bred under specific pathogen-free (SPF) or germ-free conditions at Institute for Quantitative Biosciences (IQB), The University of Tokyo. Mice were male, 15–16-week-old AID knockout mice with BALB/c background and housed in plastic isolators for human intestinal microbiota transplantation. All animal experiments were conducted in compliance with the Regulations for the Conduct of Animal Experiments of The University of Tokyo and the Regulations of the Committee on Animal Experiments of IQB.

### Fecal transplantation, IgA treatment, and DSS-induced colitis

AID-deficient SPF mice were administered with a mixture of antibiotics including metronidazole (Wako, 0.05 mg/g/day), gentamicin (nacalai, 0.5 mg/g/day) and kanamycin (Wako, 0.15 mg/g/day) for 4 days by gavage (day -4 to -1). Mice were administrated with healthy control or IBD patient stool sample by gavage (day 0). Mice were treated with rW27 or W37 (100 µg/day/mouse) by gavage for seven consecutive days (day 7 to 14). For studies in dextran sodium sulfate (DSS)-induced colitis, mice were administered with 5% DSS (molecular weight, 40,000 to 50,000; MP Biomedicals) ad libitum from day 10 to 14. Feces score was estimated based on criteria described as a previous study [17].

### Measurement of mouse fecal Lipocalin-2

Mouse feces samples were weighted, suspended in 9 volumes of PBS, and centrifuged (50 g, 15 min, 4 °C) to remove large particles. Then feces samples were centrifuged (8,000 g, 5 min, 4 °C) and supernatants were collected to measure lipocalin 2. The lipocalin 2 levels were quantified using Mouse Lipocalin-2 ELISA Kit (abcam) according to the manufacturer’s instructions.

### Statistical analysis

Statistical analyses were performed using GraphPad Prism version 9.5.1 for Windows (GraphPad Software) and the vegan package for R (version 2.6–4) to calculate PERMANOVA (adonis). Differences between two individual groups were compared using a two-tailed unpaired Student’s t test, Mann–Whitney U test, and chi-squared test. For groups of three or more, normal distribution test was performed by the Shapiro–Wilk test. Then, one-way analysis of variance (ANOVA) followed by Tukey's multiple comparison test or Kruskal–Wallis test with Dunn’s multiple comparison test was performed depending on the distribution. The correlation statistical analysis was performed by Pearson correlation coefficients. The statistical test and details about group number and replicates are indicated in the figure legends. P value was indicated in each figure.

## Results

### The difference in gut *bacteria* between healthy controls and IBD patients

**Fig. 1:**
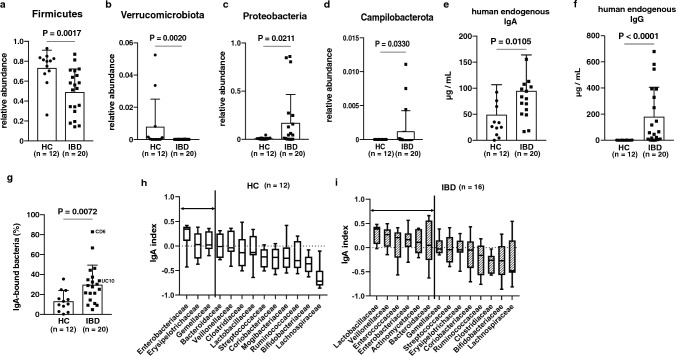
IBD patients have aberrant IgA to gut bacteria. **a–d** Relative abundances of **a** Firmicutes, **b** Verrucomicrobiota, **c** Proteobacteria, and **d** Campylobacterota in healthy controls (HC) (*n* = 12) and IBD patients (*n* = 20). **e–f** The concentration of fecal human endogenous IgA and IgG in HC (*n* = 12) and IBD patients (*n* = 20, UC: *n* = 13, CD: *n* = 7). **g** Frequency of human endogenous IgA-bound bacteria in gut bacteria of HC (*n* = 12) and IBD patients (*n* = 20). **h–i** Human endogenous IgA index of **h** HC (*n* = 12) and **i** IBD patients (*n* = 16). Statistical analysis was performed by (a-g) by Mann–Whitney U test. Data are expressed as mean ± s.d. in (**a–g**), and median ± range in (**h–i**)

We first analyzed whether IBD patients have different gut microbiota compared with healthy controls. Information on IBD patients (n = 20) and healthy controls (n = 12) is indicated in Table 1 and Supplementary Table 1. By 16S rRNA sequencing, we observed a significantly different diversity of microbiota between healthy controls and IBD patients (Supplementary Fig. 1a-b). In the case of phylum level, the composition of microbiota was different between healthy controls and IBD patients. IBD patients showed an increase in Proteobacteria and Campylobacterota and a decrease in Firmicutes and Verrucomicrobiota, especially Firmicutes, which was significantly reduced in both UC and CD patients. (Fig. 1a-d, Supplementary Fig. 1c-d). In the analysis of the amplicon sequencing variant (ASV) levels, there was no significant difference because the microbiota differed among individuals (Supplementary Fig. 2a). To analyze more details of the difference in microbiota composition between the two groups, we performed Lefse analysis. The increase　of Enterobacteriaceae and Campylobacterota and the decrease of Lachnospiraceae and Verrucomicrobiota were confirmed (Supplementary Fig. 2b). Other articles also reported that these bacteria are enriched in IBD patients [3, 25, 26]. These results indicate that our samples represent a widespread dysbiosis in IBD.

### Disorder of endogenous IgA binding ability to microbiota in IBD patients

Because IgA plays a pivotal role in the regulation of gut microbiota, we hypothesized that dysbiosis in IBD patients is caused by aberrant production of intestinal IgA in either quantity or quality. Therefore, we measured the concentration of fecal endogenous IgA and IgG in IBD patients and healthy controls. The fecal IgA and IgG were significantly increased in IBD patients compared with healthy controls, indicating enough amount of antibody production in IBD patients of this study (Fig. 1e-f). Fecal IgG production, but not IgA, is significantly increased in UC patients (Supplementary Fig. 3a). Next, we examined the relationship between antibody level and the severity score of UC patients (Mayo score) or of CD patients (CD activity index; CDAI). The correlation between antibody levels and severity score could only be observed in fecal IgG levels vs. Mayo score in UC patients, but not other correlations (Supplementary Fig. 3b-c), as reported previously [27, 28].

Since the quality of IgA is important to maintain gut homeostasis [13, 17], we checked whether endogenous IgA in the two groups has different binding ability against gut bacteria. In addition to the increasing quantity of fecal IgA, the proportion of endogenous IgA-bound bacteria was also significantly increased in IBD patients compared to healthy controls (Fig. 1g, Supplementary Fig. 3d). In UC patients, the proportion of IgA-bound bacteria and the severity score have a strong correlation, but not in CD patients (Supplementary Fig. 3e). These results suggest that the binding ability of endogenous IgA is helpful to evaluate the severity of UC patients, but not of CD patients.

Next, we analyzed what kinds of bacteria were trapped by endogenous IgA. We sorted endogenous IgA-bound and -unbound bacteria (Supplementary Fig. 4a). In healthy controls, we could observe a significant difference in β-diversity, but not in α-diversity (Supplementary Fig. 4b-c). Lefse analysis revealed that Enterobacteriaceae was enriched in IgA-bound bacteria (Supplementary Fig. 4d). In IBD patients, however, there was no significant difference in diversity analysis (Supplementary Fig. 4e-f). In Lefse analysis in IBD patients, endogenous IgA bound not only to Enterobacteriaceae but also to Lactobacillaceae (Supplementary Fig. 4 g), indicating aberrant binding ability of intestinal IgA derived from IBD patients.

Because Lefse analysis could not completely reflect individual microbiota, we performed IgA-seq to evaluate IgA-bound (IgA index > 0) and -unbound (IgA index = 0 or < 0) bacteria in individuals more precisely. In healthy controls, their endogenous IgA strongly bound to Enterobacteriaceae in IgA-seq (Fig. 1h). However, in IBD patients, their endogenous IgA bound most strongly to Lactobacillaceae, in addition to Veillonellaceae, Enterobacteriaceae (Fig. 1i, Supplementary Fig. 4 h-i). These results indicate that IBD patient-derived endogenous IgA has a disordered binding ability to microbiota compared with healthy control-derived ones and that this disorder of IgA quality may cause dysbiosis in IBD patients.

### Mouse IgA binds to human colitogenic gut *bacteria*

Because IBD patient-derived endogenous IgA has an aberrant binding ability to gut bacteria, we hypothesized that oral treatment of mouse IgA, which binds selectively to colitogenic bacteria, may ameliorate dysbiosis in IBD patients. To prove our hypothesis, we selected six mouse IgA clones (rW27, W37, rPG151A, rPG160A, PGSI1A, and SPFSI12), which bind to both *E. coli* and *C. difficile* (Supplementary Fig. 5a-d).

**Fig. 2:**
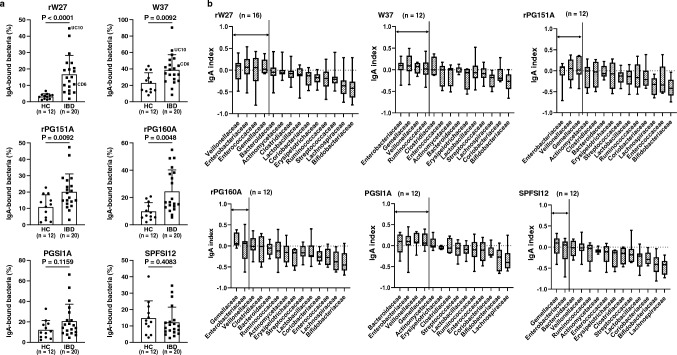
Mouse IgA antibodies bind to human colitogenic bacteria. **a** Frequency of mouse IgA-bound gut bacteria in HC (*n* = 12) and IBD patients (*n* = 20). **b** Mouse IgA index of IBD patients (rW27 (*n* = 16), W37, rPG151A, rPG160A, PGSI1A, and SPFSI12 (*n* = 12)). Statistical analysis was performed by **a** Mann–Whitney U test. Data are expressed as mean ± s.d. in (**a**), and median ± range in (**b**)

We addressed the question of whether these mouse IgA bind to human microbiota. As shown in Fig. 2a, all six mouse IgA bound to gut bacteria of both healthy controls and IBD patients. Four clones (rW27, W37, rPG151A, and rPG160A) showed significantly high-binding ability to microbiota from IBD patients compared with healthy controls (Fig. 2a, Supplementary Fig. 6a). We next sorted and analyzed mouse IgA-bound and -unbound bacteria in IBD patients. Diversity analysis was not significantly different (Supplementary Fig. 6b-c). Next, we performed Lefse analysis between mouse IgA-bound and -unbound bacteria. The rW27, W37, and PGSI1A showed a high binding ability to Enterobacteriaceae (Supplementary Fig. 7). In addition, rW27 clearly showed a weak binding ability to Bifidobacteriaceae, indicating that rW27 is the best to distinguish harmful from beneficial bacteria in human gut bacteria (Supplementary Fig. 7). In contrast to rW27, rPG150A, rPG160A, and SPFSI12, showed weak binding to Actinobacteriota but did not bind to harmful bacteria (Supplementary Fig. 7).

To assess more details of the binding ability of each antibody to gut bacteria, we performed IgA-seq (Fig. 2b, Supplementary Fig. 8a-b). IgA-seq revealed that all six mouse IgA showed high IgA index to Enterobacteriaceae and Gemellaceae, but low IgA index to beneficial bacteria, such as Lactobacillaceae and Lachnospiraceae, including short-chain fatty acid (SCFA)-producing bacteria in IBD patients (Fig. 2b). In addition, rW27 also bound to Veillonellaceae and Enterococcaceae, both of which are considered as colitogenic bacteria (Fig. 2b) [29, 30]. These results suggest that, among six IgA clones, rW27 is the best candidate IgA binding to a broad range of colitogenic bacteria including Enterococcaceae [30].

To confirm whether rW27 selectively binds to colitogenic bacteria, we next analyzed rW27-bound and -unbound bacteria in healthy controls, in which colitogenic bacteria are not enriched. Diversity analysis showed no differences in α-diversity, but a significant difference in β-diversity (Supplementary Fig. 9a-b). In Lefse analysis, Enterobacteriaceae was not detected, but Clostridiaceae was recognized as rW27-bound bacteria in healthy controls (Supplementary Fig. 9c). Furthermore, IgA-seq revealed that rW27 antibody showed weak binding (IgA index = 0 or < 0) to healthy control-derived microbiota (Supplementary Fig. 9d).

Taken together, mouse IgA, especially rW27, selectively binds to colitogenic bacteria to which IBD patients-derived endogenous IgA have not strongly bound. These results raise a possibility that oral treatment of mouse IgA ameliorates dysbiosis of IBD patients by complementing the aberrant endogenous IgA secreted in IBD patients.

### Mouse IgA inhibits the growth of IgA-bound colitogenic *bacteria*

**Fig. 3:**
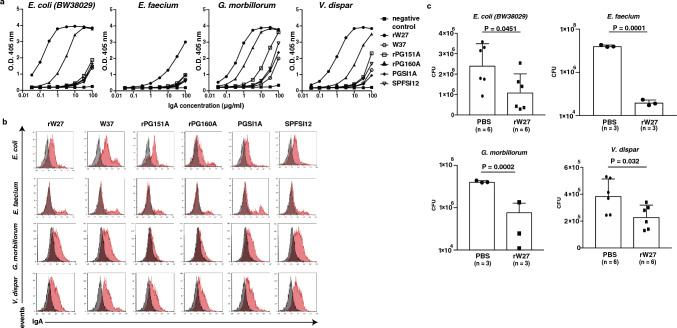
rW27 inhibits the growth of human colitogenic bacteria. (a-b) Binding analysis of indicated monoclonal IgA to *E. coli, E. faecium*, *G. morbillorum* and *V. dispar* by **a** ELISA and **b** FACS. **b** Grey histogram: unstained bacteria. Red histogram: mouse IgA-bound bacteria. **c** Growth inhibition of *E. coli* (*n* = 6)*, E. faecium* (*n* = 3), *G. morbillorum* (*n* = 3)*,* and *V. dispar* (*n* = 6) mediated by rW27. **a, b** Representative data of repeated experiments. Results of *E. coli* were same as in Supplementary Fig. 5 a and c. Statistical analysis was performed by **c** unpaired Student’s t test. Data are expressed as mean ± s.d. in (**c**)

Because our previous report [17] indicated that W27 IgA derived from hybridoma has shown growth inhibition activity against *E. coli*, we analyzed whether rW27 inhibits the growth of strongly rW27-bound bacteria, such as Enterobacteriaceae, Enterococcaceae, Gemellaceae, and Veillonellaceae (Fig. 2b). We examined the abundance of these four kinds of bacteria in IBD patient-derived microbiota and found that a particular patient had a high proportion of unique bacterial family (Supplementary Fig. 10a–d). For growth inhibition assay, we selected *E. coli, E. faecium, G. morbillorum,* and* V*. *dispar* as representative bacterial strains from each family. We first checked whether rW27 binds to these bacteria and confirmed that rW27 binds to them (Fig. 3a-b).

As reported previously [17], we tested whether rW27 inhibits the growth of them and found that rW27 significantly suppressed the growth of these kinds of bacteria (Fig. 3c). These findings suggest that rW27 may inhibit the growth of these colitogenic bacteria in the intestinal lumen.

### Mouse IgA suppress gut inflammation in gnotobiotic mice with IBD patients-derived microbiota

The rW27 showed a high binding ratio to intestinal bacteria of IBD patients (Fig. 2a) and inhibited the growth of rW27-bound bacteria (Fig. 3c). Therefore, we investigated whether oral administration of mouse IgA to gnotobiotic mice with IBD patient-derived microbiota could improve intestinal dysbiosis and influence susceptibility to colitis. Because normal endogenous IgA in wild-type mice affect the gut bacteria, we used AID-deficient mice [9, 10], which do not produce IgA in the gut. We inoculated mice with IBD patient-derived microbiota (UC10) with the highest rW27 binding proportion (44.7%) to clearly evaluate the effect of rW27 (Fig. 2a, Supplementary Fig. 6a). Considering that there are differences in the effect between antibodies due to the different microbiota in each patient, a comparative study was conducted with W37, which also showed a high binding ratio (62.5%) to UC10 microbiota (Fig. 2a, Supplementary Fig. 6a).

**Fig. 4:**
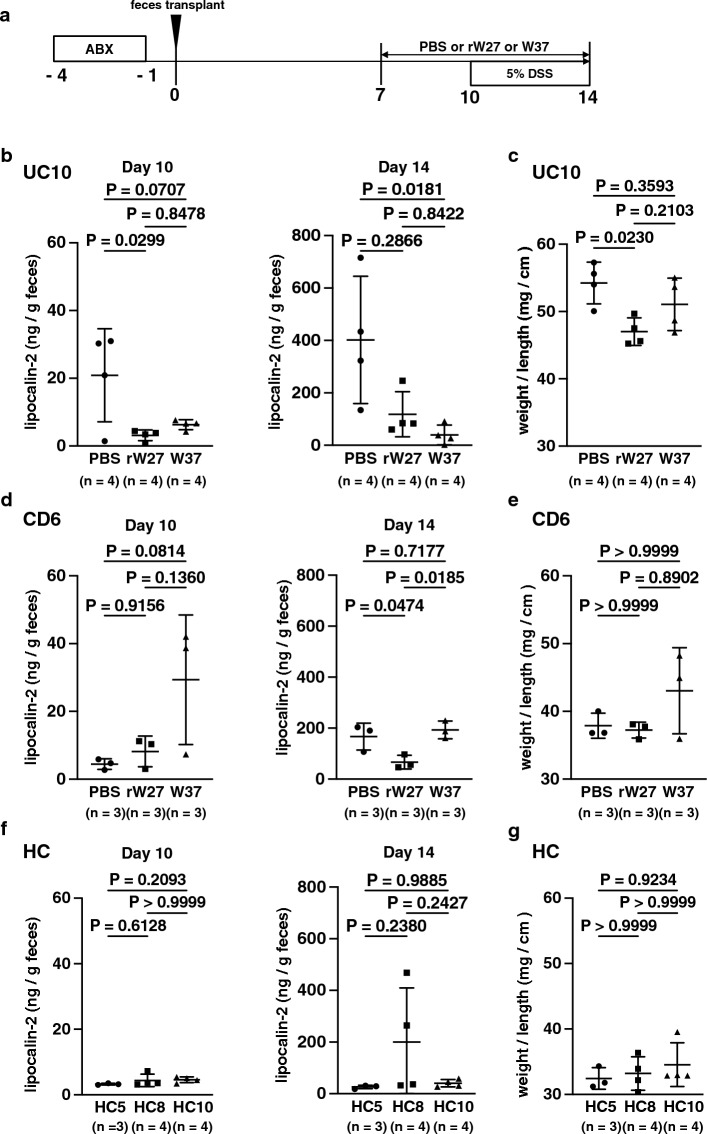
The rW27 oral treatment ameliorates gut inflammation in gnotobiotic mice with IBD patients-derived gut bacteria. **a** Schedule of fecal transplantation, IgA and DSS treatments. **b, c** **b** Concentration of fecal lipocalin 2 at day 10 and day 14, **c** The colon weight/length ratio in gnotobiotic mice with UC10-derived microbiota (n = 4), **d, e** **d** Concentration of fecal lipocalin 2 at day 10 and day 14, **e** The colon weight/length ratio in gnotobiotic mice with CD6-derived microbiota (n = 3). **f, g** **f** Concentration of fecal lipocalin 2 at day 10 and day 14, **g** The colon weight/length ratio in healthy control-derived microbiota (HC5: *n* = 3, HC8: *n* = 4, HC10: *n* = 4). Statistical analysis was performed by **b–g** Kruskal–Wallis test with Dunn’s multiple comparison test or one-way ANOVA with Tukey's multiple comparisons test. Data are expressed as mean ± s.d. in (**b–g**)

After inoculated with UC10 microbiota, mice were orally administered with mouse IgA. During the oral administration period of antibody, mice were induced colitis with DSS, and the sensitivity of colitis was evaluated (Fig. 4a). We could not observe any significant difference in body weight change and feces score among three groups (Supplementary Fig. 11a). Therefore, to evaluate the low levels of inflammation in the colon, we measured the concentration of fecal lipocalin 2 as an inflammation marker before (day 10) and after (day 14) DSS treatments. Interestingly, orally treated mice with both rW27 and W37 showed no increase of fecal lipocalin 2 compared with the untreated group (Fig. 4b). The weight/length ratio of the colon is also lower in the mouse IgA-treated groups than in the untreated group (Fig. 4c). In addition, we inoculated mice with other IBD patient-derived microbiota (CD6) with the highest endogenous IgA binding proportion (88.2%, Fig. 1g, Supplementary Fig. 3d). In these mice, the increase in lipocalin 2 levels after DSS in the rW27 group was suppressed compared with the other groups (Fig. 4d-e, Supplementary Fig. 11b). Compared to mice inoculated with IBD patients-derived microbiota, mice inoculated with healthy control-derived ones had little increase in lipocalin 2 (Fig. 4f-g, Supplementary Fig. 11c). These findings suggest that susceptibility of DSS-induced colitis was reduced by oral treatment of rW27.

### Mouse IgA remedy dysbiosis in gnotobiotic mice with IBD patients-derived microbiota

**Fig. 5:**
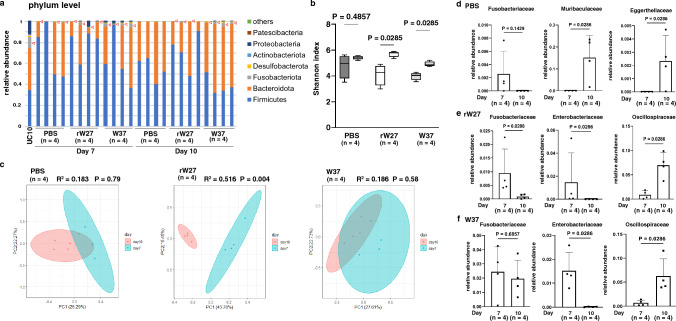
Mouse IgA antibodies modulate gut bacteria of gnotobiotic mice with IBD patient-derived gut bacteria. **a** Relative abundances of bacterial taxa at phylum level in UC10 patient gut microbiota, PBS-, rW27-, and W37-treated mice at day 7 and day 10 (*n* = 4). Red arrows indicate Fusobacteriaceae. **b** Shannon index of PBS-, rW27-, and W37-treated mice at day 7 (left bar) and day 10 (right bar) (*n* = 4). (c) Unweighted unifrac distance of PBS-, rW27-, and W37-treated mice at day 7 (blue circle) and day 10 (red circle) (*n* = 4). **d–f** Relative abundance of indicated bacteria of **d** PBS-, **e** rW27-, and **f** W37-treated mice at day 7 and day 10 (*n* = 4). Statistical analysis was performed by **b, d–f** by Mann–Whitney U test and **c** PERMANOVA comparison. Data are expressed as median ± range in (**b**) and mean ± s.d. in (**d–f**)

Because oral administration of mouse IgA suppressed intestinal inflammation, we hypothesized that antibody administration affects gut microbiota in gnotobiotic mice. We compared microbiota before (day 7) and after (day 10) IgA administration. While the microbiota of UC10 patient contained Fusobacteriota and Proteobacteria, Firmicutes and Bacteroidota were predominant in gnotobiotic mice, indicating the transplanted microbiota could not be reproduced completely in the mouse intestine (Fig. 5a, Supplementary Fig. 12a-b). Only limited bacteria from donor’s microbiota were reproduced in mice inoculated with CD6- or healthy control-derived gut bacteria (Supplementary Fig. 12c-d). Since Fusobacteriaceae were not originally present in mice intestine, they were usually difficult to colonize. However, Fusobacteriaceae were detected in 7 of 12 animals in the gut on day 10, indicating a partial value for UC10 fecal transplantation experiment (Fig. 5a, Supplementary Fig. 12a). Comparison of α-diversity between day 7 and day 10 showed the significant increase in rW27- and W37-treated group, but not in the untreated group (Fig. 5b). In addition, β-diversity was significantly changed at day 10 compared with day 7 in the rW27-treated group, indicating that mouse IgA affected gut microbiota (Fig. 5c).

In the untreated group, Fusobacteriaceae was colonized at day 7 but not detected at day 10 because the abundance of this bacteria was low (Fig. 5d). In Lefse analysis, there was a significant increase in Eggerthellaceae and Muribaculaceae, which were reported as inflammation-inducing bacteria (Fig. 5d, Supplementary Fig. 13a) [31, 32]. In contrast, the rW27-treated group showed that an increase in Oscillospiraceae including SCFA-producing bacteria and a decrease in Enterobacteriaceae and Fusobacteriaceae (Fig. 5e, Supplementary Fig. 13b), indicating that rW27 administration improved dysbiosis. Although W37 reduced fecal lipocalin 2, Fusobacteriaceae was not changed between day 7 and day 10 (Fig. 5f, Supplementary Fig. 13c). However, an increase of Oscillospiraceae and a decrease of Enterobacteriaceae were also observed in the W37-treated group (Fig. 5f, Supplementary Fig. 13c). The different effects of rW27 and W37 on human microbiota suggest that an appropriate selection of antibodies is necessary to remedy dysbiosis in individual patient.

## Discussion

In this study, we revealed that IBD patients produce endogenous IgA which has an aberrant binding ability to microbiota. Therefore, oral supplementation of mouse IgA was effective to modulate microbiota and suppressed gut inflammation in gnotobiotic mice with IBD patient-derived microbiota. The effect on gut bacteria was different between rW27 and W37 treatment, indicating that it is important to select a suitable mouse IgA clone for individual patient to maintain healthy microbiota.

Recent studies demonstrated that IBD is associated with dysbiosis [2]. In previous studies, IL-10 or IL-2 deficient mice spontaneously develop colitis in a gut bacteria-dependent manner [33, 34]. These mice also showed dysbiosis of microbiota [35]. Although accumulating evidence suggests that dysbiosis is associated with the development and pathogenesis of colitis, it remains unclear what factor induces dysbiosis. Our finding demonstrates a possibility that IBD patients produce an inappropriate IgA to interact with gut bacteria, resulting in dysbiosis with increased pathobiont and colitis in IBD patients. However, it remains to address the question of what factor induces the production of aberrant IgA in IBD patients.

Although it has been suggested that colitogenic bacteria play a major role in the development of colitis, there is no effective treatment for gut microbiota. In recent years, fecal microbiota transplantation (FMT) and live biotherapeutic products (LBP) have been developed as a new treatment to control microbiota in IBD patients. However, since the definition of a healthy donor has not been established, it is difficult to ensure the safety of bacteria and the stable bacterial colonization to patients’ gut lumen. Therefore, an additional strategy to improve gut microbiota is needed worldwide.

In our approach, oral treatment of appropriate IgA to gut lumen can compensate the problem of FMT and LBP, since IgA selectively targets colitogenic bacteria, but not beneficial bacteria. More importantly, our study demonstrated that supplemented IgA is not necessarily human-derived IgA, since mouse IgA showed the selective binding ability to human gut bacteria and significantly modulated microbiota of gnotobiotic mice. Our findings agree with the previous study that W27 controls mouse gut microbiota [13, 17, 18] and modulates human intestinal microbiota in vitro [19]. To modulate gut microbiota of IBD patients whose endogenous IgA has the aberrant binding ability to gut bacteria, recombinant mouse IgA can provide a stable supply and ensure safety, which has been difficult with FMT and LBP.

The DSS-induced colitis model has been used in many studies as IBD model because dysbiosis has also occurred. Our results indicated that mouse IgA, especially rW27, not only controlled the gut microbiota but also suppressed the increase in lipocalin 2 level, which is a significant finding that raises the possibility of treatment of IBD. However, the limitation of this study is the difficulty in reproducing the gut microbiota of IBD patients in mice because the intestinal environment of mice is different from that of human. Because the susceptibility to DSS varies with the gut microbiota, mice inoculated with healthy control- or CD6-derived microbiota may not develop severe colitis. Our results suggest that there are not only dysbiosis but also immune disorders in IBD patients. In this study, AID-deficient mice did not show robust colitis by simply transplanting gut microbiota. To overcome these limitations, various approaches are necessary, such as examination of mice with abnormal immunity close to IBD patients including IL-10-deficient mice. In the future, we hope that not only a single clone but also a combination of mouse IgA will be used to regulate gut microbiota for clinical in patient.

### Supplementary Information

Below is the link to the electronic supplementary material.

Supplementary file1 (XLSX 15 KB)

Supplementary Figure 2 (EPS FILE 1,113 KB)

Supplementary Figure 3 (EPS FILE 10,431 KB)

Supplementary Figure 4 (EPS FILE 1,081 KB)

Supplementary Figure 5 (EPS FILE 4,665 KB)

Supplementary Figure 6 (EPS FILE 8,747 KB)

Supplementary Figure 7 (EPS FILE 4,151 KB)

Supplementary Figure 8 (EPS FILE 1,481 KB)

Supplementary Figure 9 (EPS FILE 1,362 KB)

Supplementary Figure 10 (EPS FILE 3,193 KB)

Supplementary Figure 11 (EPS FILE 881 KB)

Supplementary Figure 12 (EPS FILE 889 KB)

Supplementary Figure 13 (EPS FILE 18,761 KB)

Supplementary Figure 14 (EPS FILE 1,663 KB)
